# Effect of transient scrotal hyperthermia on human sperm: an iTRAQ-based proteomic analysis

**DOI:** 10.1186/s12958-020-00640-w

**Published:** 2020-08-12

**Authors:** Yan-Qing Wu, Meng Rao, Shi-Fu Hu, Dan-Dan Ke, Chang-Hong Zhu, Wei Xia

**Affiliations:** 1 Institute of Reproductive Health, Tongji Medical College, Huazhong University of Science and Technology, Wuhan, People’s Republic of China; 2 Department of reproduction and genetics, the first affiliated hospital of Kunming medical university, Kunming, People’s Republic of China; 3 Department of Obstetrics and Gynecological Ultrasound Imaging, Renmin Hospital of Wuhan University, Wuhan, People’s Republic of China; 4 Reproductive Medicine Center, Tongji Medical College, Huazhong University of Science and Technology, Wuhan, People’s Republic of China

**Keywords:** Sperm, Proteomics, ITRAQ, Hyperthermia

## Abstract

**Background:**

Through this prospective study, we aimed to explore the change of molecular modification after the transient scrotal hyperthermia on human sperm.

**Methods:**

Ten healthy subjects selected with strict screening criteria underwent testicular warming in a 43 °C water bath for 30 min a day for 10 consecutive days. Semen samples were collected 2 weeks before the first heat treatment and 6 weeks after the first heat treatment. Proteins from the samples were labeled with isobaric tags for relative and absolute quantitation and analyzed by two-dimensional liquid chromatography–tandem mass spectrometry.

**Results:**

In contrast to the control, of the 3446 proteins identified, 61 proteins were deregulated: 28 were up-regulated and 33 were down-regulated. Approximately 95% of the differentially expressed proteins were found to participate in spermatogenesis, fertilization, or other aspects of reproduction. In particular, the expression of sperm motility and energy metabolism-related proteins AKAP4, SPESP1, ODF1, ODF2, GAPDHS, and ACTRT2, validated by western blotting of the proteins obtained from human and mouse samples, tended to be reduced under scrotal hyperthermia.

**Conclusions:**

The results indicated that the proteins AKAP4, ODF1, ODF2, GAPDHS, SPESP1, and ACTRT2, play an important role in the heat-induced reversible reduction in sperm concentration and motility and have the potential to be the biomarkers and clinical targets for scrotal heat treatment induced male infertility.

## Introduction

In mammals, including humans, the scrotal temperature required for normal spermatogenesis within the testes is 2 °C to 8 °C lower than the core body temperature. Some patients with varicocele and people in certain occupations, such as boilermakers, cab drivers, and sedentary people, may develop scrotal hyperthermia, resulting in low sperm concentration and mobility and, consequently, infertility. However, once the influencing factor disappears and the scrotal temperature returns to 33 °C–35 °C, spermatogenesis, and sperm parameters revert to normal after one or two spermatogenic cycles [1–3].

Exposing the testes of mice [4], rats [5, 6], monkeys [7], bulls [8], sheep [9], and humans [10, 11] to high temperatures induced germ cell apoptosis in spermatogenesis [12]. Heat stress in the scrotum induced the apoptosis of germ cells [13–16], imbalances in antioxidant levels leading to oxidative stress [17–19], and deregulation in the gene expression pattern [20]. Comparative proteomics of testis biopsy specimens of men subjected to transient scrotal hyperthermia has revealed that these deregulated proteins were associated with germ cell proliferation and apoptosis [21]. Heat stress not only destroys spermatocytes but also affects sperm maturation in the caput epididymis [22]. Exposing sperm to hyperthermia in vitro has been shown to reduce total sperm motility and metabolic activity [23]. In a previous study, we found that oxidative stress, mitochondrial dysfunction, and tight packaging of sperm DNA could induce reversible impairment of spermatogenesis [24]. We also found that sperm concentration and motility in human subjects who experienced scrotal warming in a 43 °C water bath for 30 min a day for 10 consecutive days reduced to the lowest levels (30 and 65%, respectively) in the sixth week after treatment [24, 25]. Although many studies have evaluated the effect of scrotal hyperthermia, the molecular change underlying the oligospermia or asthenospermia induced by heat stress remains unclear.

To understand this molecular modification, we used isobaric tags for relative and absolute quantitation (iTRAQ)-based proteomic analysis to explore the potential effects of scrotal temperatures on male reproductive function. Here we show that these deregulated proteins mainly participate in spermatogenesis, fertilization, and reproduction, of which these expressions of down-regulated proteins AKAP4, ODF1, ODF2, GAPDHS, SPESP1 and, ACTRT2 were validated by western blotting of proteins obtained from human and mouse sperm samples.

## Material and methods

### Subjects and human sperm specimens

This study was approved by the Ethics Committee of Tongji Medical College, Huazhong University of Science and Technology. Subjects were recruited using the same method and following the same exclusion criteria as in our previous study [25]. Briefly, men aged between 22 and 50 years, who had at least one child were included, and those with cryptorchidism or varicocele, without children, or with severe heart, brain, or renal disease or with sexually transmitted infections, epididymitis, epididymo-orchitis in anamnesis were excluded [26]. In addition, participants were instructed do not to keep in trousers pockets cell phones during the study because of their radiation negative influence on semen quality [27]. Ten subjects met our criteria, of which the subjects underwent testicular warming in a 43 °C water bath for 30 min a day for 10 consecutive days. The lower half body of each subject was immersed in the bathtub with the water regulated to 43 °C. To keep the water temperature constant, we continuously added the adjusted hot water (43 °C) into the bathtub. Meanwhile, we also drained the water from the bathtub by the same flow rate. Sperm specimens were collected 2 weeks before and 6 weeks after the first heat treatment and were prepared for proteomic analysis by iTRAQ and two-dimensional liquid chromatography–tandem mass spectrometry (2D-LC–MS/MS) to identify differentially expressed proteins.

### Heat treatment in mice

Fifty adult male ICR mice (8–10 weeks old) were obtained from the Center for Disease Control and Prevention of Hubei Province in China (Grade SPF, Certificate No. SCXK 2015–0018). All mice were fed and maintained under a controlled environment of 20 °C–22 °C, 12/12-h light/dark cycle, and 60% humidity, with ad libitum access to food and water. All animal procedures were approved by the Institutional Review Board of Tongji Medical College and were performed by the National Institutes of Health Guiding Principles on the Care and Use of Animals. The mice were anesthetized with intraperitoneal injections of sodium pentobarbital at 30–50 mg/kg body weight. As described previously [28], the anesthetized mice were placed on a handmade raft in a posture that kept the scrotum immersed in water for 30 min at 43 °C (heat treatment group) or 33 °C (control). The mice were then transferred to a warm room until recovery from anesthesia.

Mature sperm samples were obtained from the epididymis of the mice as previously described [28]. Briefly, sperm samples were obtained by the oil drop method 21 days after the heat treatment, which is when the sperm concentration and motility after hyperthermia were considered to be lowest.

### Protein sample preparation and iTRAQ labeling

The human sperm samples were washed three times with phosphate-buffered saline (PBS) and ground to the powder with liquid nitrogen. The total proteins in the control group (sperm specimens collected 2 weeks before the first heat treatment) and heat group (sperm specimens collected 6 weeks after the first heat treatment) were dissolved in protein extraction solution [9 M urea, 4% CHAPS, 1% DTT, 1% IPG buffer (GE Healthcare)] at 30 °C for 1 h. The reaction liquid was then centrifuged at 15,000×*g* for 15 min at room temperature, and the supernatant was collected and analyzed by the Bradford method to determine the protein concentration. Equal amounts of each protein sample were dissolved and reacted with reductive alkylation and digested with trypsin according to the iTRAQ instructions. The digested peptides were collected and incubated with iTRAQ reagent (SCIEX, USA). Two biological replicates for before and after heat treatment samples were prepared for both groups. The replicates of one group were labeled with isobaric tags 113 and 114, and those of the other group were labeled with tags 114 and 115. The freeze-dried samples being treated by nitrogen were dissolved in Buffer A solution, and SCX chromatography was performed using an Agilent 1200 HPLC System (Agilent) using the following parameters: Poly-SEA, 5 μ, 300 Å, 2.0 × 150 mm, 0.5-ml/min flow, and UV detection at 215 nm and 280 nm. Mixed labeled proteins were separated into 12 segments by liquid chromatography. The data were acquired using a Triple TOF 5600 System (SCIEX, USA) together with a Nanospray III source (SCIEX, USA) and a pulled quartz tip as the emitter (New Objectives, USA), with the ion spray voltage at 2.5 kV, the curtain gas pressure at 30 psi, the nebulizer gas pressure at 5 psi, and the interface heater temperature at 150 °C.

### Protein identification and quantification

Protein data were analyzed using ProteinPilot Software v.4.5 (SCIEX, USA), which uses the Paragon algorithm for database searching against the human database. The parameters were as follows: TripleTOF 5600, iTRAQ 4-plex quantification, cysteine modified with iodoacetamide, biological modification selected as ID focus, and trypsin digestion. Using an automated bait database search strategy, the false discovery rate (FDR; i.e., false-positive matches divided by total matches) was evaluated using Proteomics System Performance Evaluation Pipeline Software integrated with ProteinPilot Software. The iTRAQ 4-plex was then selected for protein quantification with unique peptides during the search. The peptides of the sperm samples before and after heat treatment were labeled with isobaric tags 113/115 and 114/116, respectively. One biological replicate of each before and after heat treatment samples was again labeled with isobaric tags 115 and 116. The isobaric tag-labeled samples were then pooled. All proteins with FDR < 1% and the number of peptides > 1 were further analyzed. The differentially expressed proteins were determined by *p-*values based on the ratios of different protein markers. The proteins that conformed to the requirements of a mean fold change of > 1.5 or < 0.67 and a *p*-value of < 0.05 were considered to be differentially expressed.

### GO and KEGG pathway enrichment analysis

All proteins of interest were matched by a homology search against the human sperm protein database for gene ontology (GO) and KEGG pathway enrichment analysis. The linkages between the protein ID and GO information were established using the public databases NCBI, KEGG, and GO. GO analysis was performed using the Quick GO database, and statistical methods, including the Fisher’s exact test, were used to obtain *p* values. The annotations for pathways of enriched proteins were used as query data for KEGG pathway enrichment analysis. The protein-protein interactions (PPIs) were identified from the String database. All annotations were associated with their information code.

### Western blotting

Sperm samples were homogenized in 80 μl of an ice-cold lysis buffer containing a cocktail of protease inhibitors and lysed by sonication several times. These homogenates were centrifuged at 12,000×*g* for 15 min at 4 °C, and the supernatant was collected for the quantification of total proteins. Samples with equal protein quantity (50 μg/lane) were electrophoresed on a 10% SDS-PAGE gel and transferred to polyvinylidene difluoride membranes (Beyotime, China). The membranes were blocked with 5% milk for 1 h at room temperature and then incubated with primary antibodies overnight at 4 °C. After washing with Tris-buffered saline with Tween 20, the membranes were incubated with secondary antibodies for 1 h at room temperature. Antibodies are listed in Supplementary Table 1. The band intensities were quantified by densitometry using ImageJ analysis software (Research Services Branch).

### Statistical analysis

Data were analyzed using the Student’s *t*-test or the Mann–Whitney *U* test in case of differences in variance (Fisher’s exact test). All analyses were performed using SPSS 19.0 and GraphPad Prism 7.0. The results are presented as the mean ± SEM and were considered significant for *p* values of < 0.05. Asterisks indicate: *, *p* < 0.05.

## Results

### Experimental design and workflow for the identification of altered proteins in human sperm before and after heat treatment

**Fig. 1:**
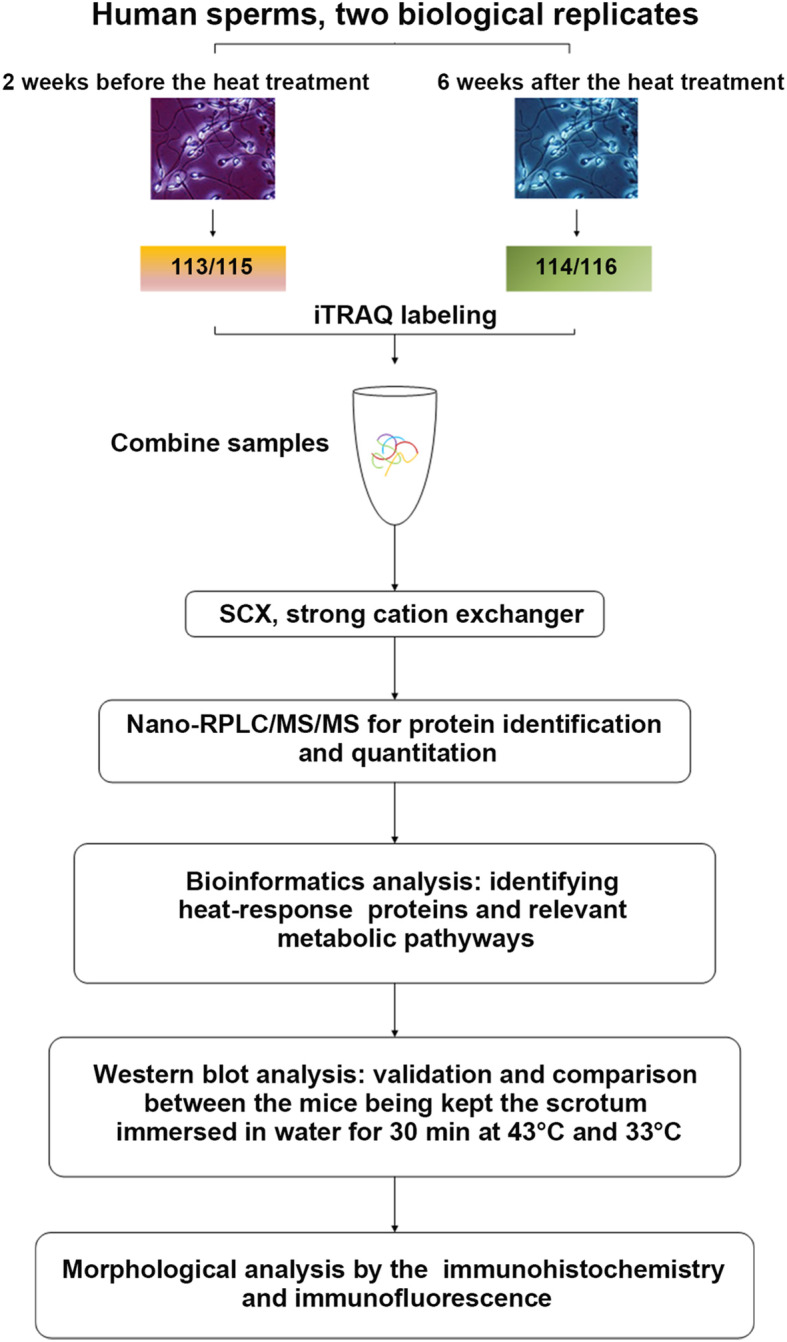
Experimental design and the workflow in this study. Two sets of biological replicate samples were analyzed by iTRAQ, using the hpRP-nanoLC-MS/MS workflow for examining proteome changes between human sperms of 2 weeks before the first treatment (control) and 6 weeks after the first local heat stress of the scrotum (Heat treatment 6w). Proteins were identified using Protein Pilot Software

To identify the heat stress-response proteins in human sperm and achieve a better understanding of the underlying metabolic process and the mechanism of spermatogenesis, we collected sperms from human sperm samples obtained 2 weeks before and 6 weeks after the first treatment and analyzed using an iTRAQ-based quantitative proteomic approach combined with 2D-LC–MS/MS (Fig. 1). The proteins in the sperm samples were quantitatively identified in two biological replicates. In total, 3446 proteins with an FDR of < 1% and a *p*-value of < 0.05 were identified (Supplementary Table 2).

### Functional characterization and annotation of the differentially expressed proteins

To define fold change values that reflect the effect of scrotal hyperthermia on human sperm, the ratios of sperm proteins 2 weeks before and 6 weeks after scrotal hyperthermia in two independent biological duplicate groups were determined by protein labeling with isobaric tags 114/113 and 116/115. In total, 139 proteins in group 1 and 135 in group 2 were found to be differentially expressed based on fold change values of > 1.50 or < 0.67. Combining the results of these two groups, 61 proteins were found to be significantly upregulated (28 proteins) or downregulated (33 proteins) in the heat-treated group 6 weeks after treatment compared with the control group (Table 1).

**Table 1:** Differentially expressed proteins in the sixth week after heat-treated group compared with those in the control group

No.	Gene	Accession	Name	Fold change (avg)
1	AKAP4	Q5JQC9	A-kinase anchor protein 4	0.60675
2	ODF2	Q5BJF6	Outer dense fiber protein 2	0.63015
3	HK1	P19367	Hexokinase-1	0.62455
4	CCIN	Q13939	Calicin	0.62445
5	GAPDHS	O14556	Glyceraldehyde-3-phosphate dehydrogenase, testis-specific	0.65205
6	TEKT5	Q96M29	Tektin-5	0.61830
7	FAM71B	Q8TC56	Protein FAM71B	0.55335
8	LRRC37B	Q96QE4	Leucine-rich repeat-containing protein 37B	0.62535
9	TPI1	P60174	Triosephosphate isomerase	0.59305
10	ODF1	Q14990	Outer dense fiber protein 1	0.60095
11	GLUL	P15104	Glutamine synthetase	0.65995
12	SPESP1	Q6UW49	Sperm equatorial segment protein 1	0.63020
13	RAB2A	P61019	Ras-related protein Rab-2A	0.62420
14	ZPBP	Q9BS86	Zona pellucida-binding protein 1	0.57560
15	ACTRT2	Q8TDY3	Actin-related protein T2	0.66370
16	RSPH9	Q9H1X1	Radial spoke head protein 9 homolog	0.65810
17	FAM71A	Q8IYT1	Protein FAM71A	0.59755
18	MENT	Q9BUN1	Protein MENT	0.55490
19	ACTL9	Q8TC94	Actin-like protein 9	0.65075
20	CYCL1	P35663	Cylicin-1	0.57220
21	CYCL2	Q14093	Cylicin-2	0.50275
22	VRK3	Q8IV63	Inactive serine/threonine-protein kinase VRK3	0.62290
23	ATP1B3	P54709	Sodium/potassium-transporting ATPase subunit beta-3	0.63620
24	FNDC8	Q8TC99	Fibronectin type III domain-containing protein 8	0.54235
25	PPIL6	Q8IXY8	Peptidyl-prolyl cis-trans isomerase-like 6	0.64450
26	SMCP	P49901	Sperm mitochondrial-associated cysteine-rich protein	0.51795
27	SLC2A5	P22732	Solute carrier family 2, facilitated glucose transporter member 5	0.65610
28	PFN3	P60673	Profilin-3	0.58565
29	TMEM190	Q8WZ59	Transmembrane protein 190	0.60795
30	SPATA31D1	Q6ZQQ2	Spermatogenesis-associated protein 31D1	0.66080
31	DYDC1	Q8WWB3	DPY30 domain-containing protein 1	0.65775
32	CD46	P15529	Membrane cofactor protein	0.63320
33	EFCAB3	Q8N7B9	EF-hand calcium-binding domain-containing protein 3	0.59870
34	HNPNPM	P52272	Heterogeneous nuclear ribonucleoprotein M	1.89130
35	CLGN	O14967	Calmegin	2.11610
36	CANX	P27824	Calnexin	1.81915
37	HNRNPA2B1	P22626	Heterogeneous nuclear ribonucleoproteins A2/B1	1.84040
38	XRCC6	P12956	X-ray repair cross-complementing protein 6	1.98985
39	DDX4	Q9NQI0	Probable ATP-dependent RNA helicase DDX4	1.72575
40	RCN2	Q14257	Reticulocalbin-2	1.67800
41	HNRNPU	Q00839	Heterogeneous nuclear ribonucleoprotein U	1.91345
42	DDX39A	O00148	ATP-dependent RNA helicase DDX39A	1.95795
43	HNRNPK	P61978	Heterogeneous nuclear ribonucleoprotein K	1.94255
44	NPM1	P06748	Nucleophosmin	1.80460
45	DDX17	Q92841	Probable ATP-dependent RNA helicase DDX17	1.76955
46	UMOD	P07911	Uromodulin	2.17185
47	HNRNPA3	P51991	Heterogeneous nuclear ribonucleoprotein A3	2.18345
48	SMC3	Q9UQE7	Structural maintenance of chromosomes protein 3	1.63640
49	RANBP1	P43487	Ran-specific GTPase-activating protein	1.70640
50	HNRNPL	P14866	Heterogeneous nuclear ribonucleoprotein L	1.74625
51	RAN	P62826	GTP-binding nuclear protein Ran	1.85995
52	DHX15	O43143	Putative pre-mRNA-splicing factor ATP-dependent RNA helicase DHX15	1.76235
53	ELAVL1	Q15717	ELAV-like protein 1	1.66060
54	HNRNPF	P52597	Heterogeneous nuclear ribonucleoprotein F	2.04530
55	KHDRBS1	Q07666	KH domain-containing, RNA-binding, signal transduction-associated protein 1	2.13890
56	HNRNPD	Q14103	Heterogeneous nuclear ribonucleoprotein D0	2.00500
57	SF3B3	Q15393	Splicing factor 3B subunit 3	1.82035
58	RBMXL2	O75526	RNA-binding motif protein, X-linked-like-2	1.60315
59	RBMY1C	P0DJD4	RNA-binding motif protein, Y chromosome, family 1 member C	2.45005
60	MYEF2	Q9P2K5	Myelin expression factor 2	1.66025
61	REG3G	Q6UW15	Regenerating islet-derived protein 3-gamma	1.68985

**Fig. 2:**
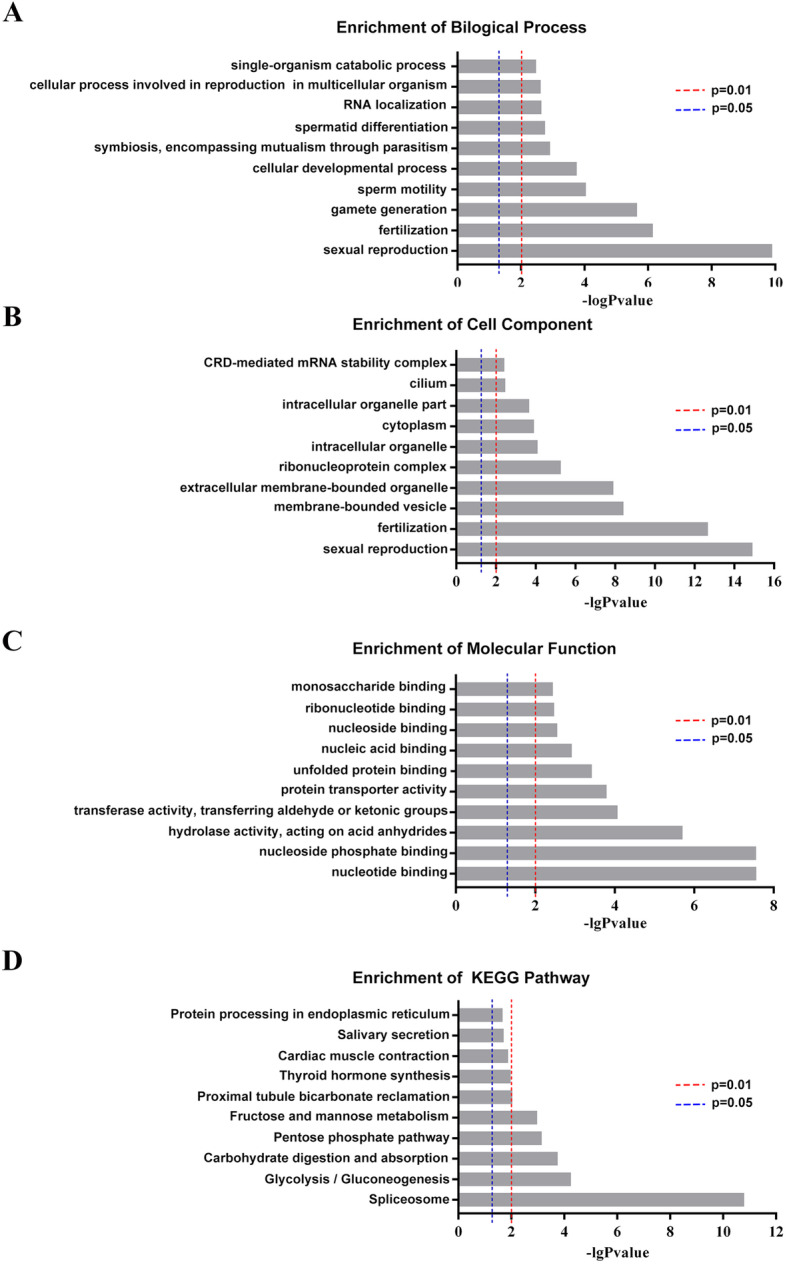
GO annotation indicated the 61 differentially expressed proteins involved the sperm motility and fertility. GO annotation in three categories (**a**: biological process (BP), **b**: cellular component (CC), **c**: molecular function (MF)), and distribution of enriched KEGG pathway (**d**). The columns are colored with a gradient from blue *(p <* 0.05) to red (*p* < 0.01)

**Fig. 3:**
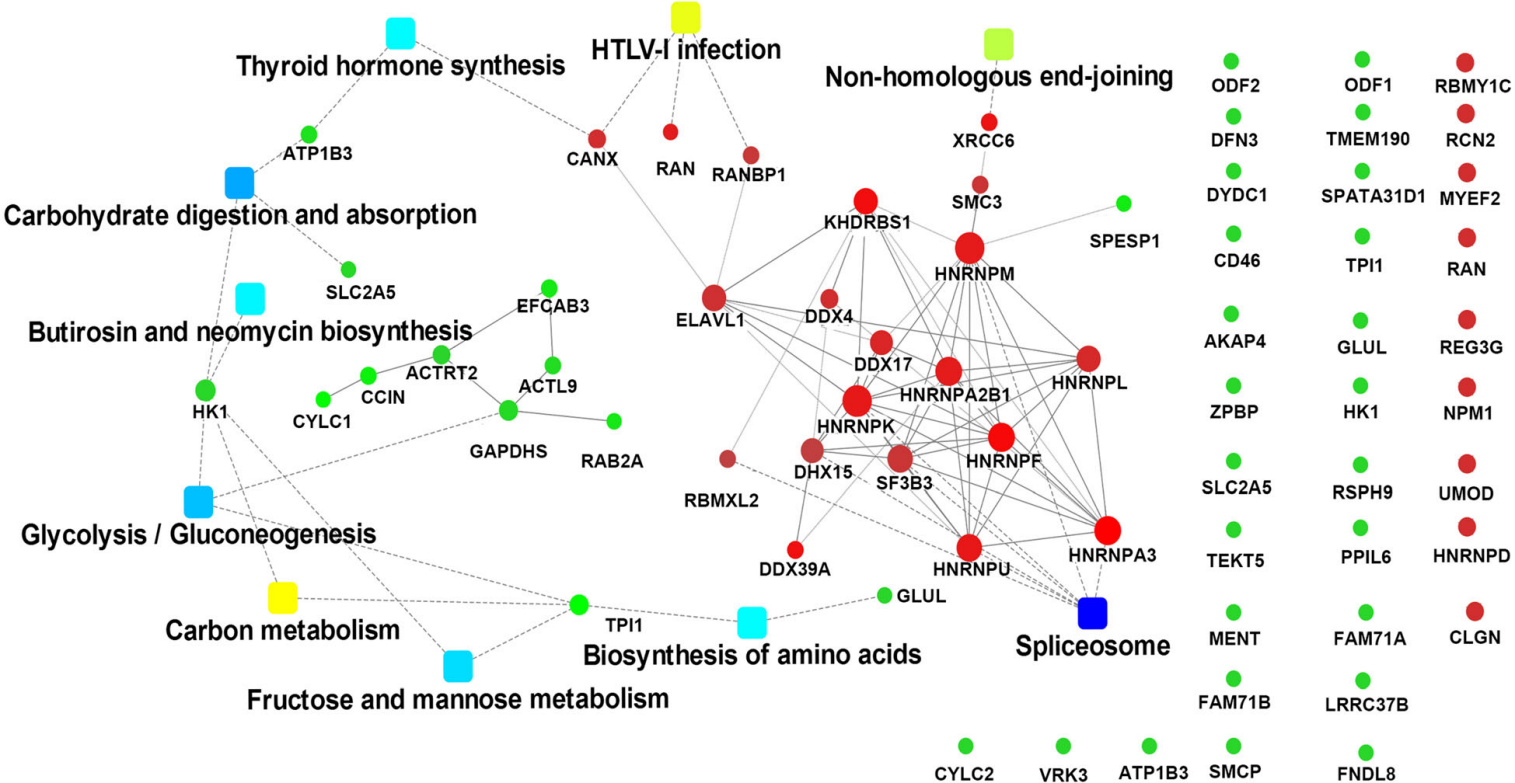
Molecular pathways of deregulated proteins involved in asthenospermia and oligospermia after heat treatment. A network of protein-protein interactions (PPI) combined with fold changes of proteins, protein-protein interactions, and KEGG pathway enrichment. Red balls represent an up-regulated protein (*p* < 0.05), green balls represent a down-regulated protein (*p* < 0.05), and green squares represent the pathway (*p* < 0.05)

To understand the functions and pathways of the differentially expressed proteins, we used the network tools provided with the DAVID, String, and KEGG databases. The identified proteins were analyzed through GO enrichment analysis (FDR *q* < 0.05, Fisher’s exact test) in the biological process (BP), cell component (CC), and molecular function (MF) categories. Sexual reproduction, fertilization, gamete generation, and sperm motility were list with most significance in BP analysis (Fig. 2a). The top 10 CC classifications of these differentially expressed proteins included intracellular organelle, ribonucleoprotein complex, cytoplasm, and cilium are shown in Fig. 2b. The top 10 MF categories are shown in Fig. 2c. KEGG analysis revealed that the most active pathways (*p* ≤ 0.05) involving the differentially expressed proteins included the splicing process, carbohydrate digestion and absorption, glycolysis/gluconeogenesis, and fructose and mannose metabolism (Fig. 2d). Probable interaction analysis of the differentially expressed proteins against the String database revealed two independent PPI networks, one containing 19 proteins and the other containing 7 proteins. The PPI networks with the top nine KEGG enriched pathways are shown in Fig. 3.

### Confirmation of heat stress-induced proteins in mouse sperm by western blotting

**Fig. 4:**
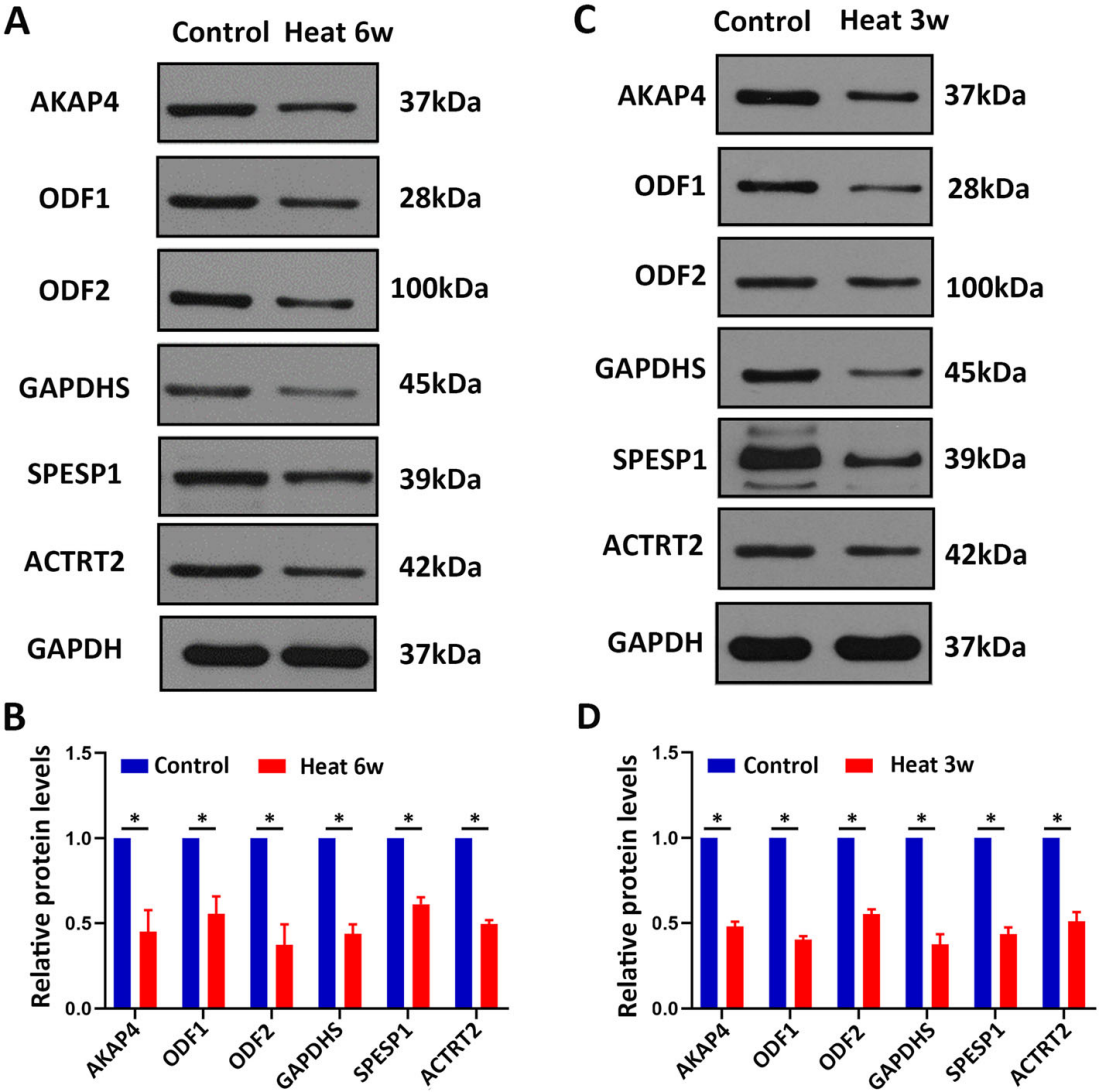
Western blot analysis showing AKAP4, ODF1, ODF2, GAPDHS, SPESP1, and ACTRT2 proteins from control and heat treatment sperm of human and mouse. **a** Western blot analysis showing AKAP4, ODF1, ODF2, GAPDHS, SPESP1, and ACTRT2 proteins from human sperms of control and heat treatment. GAPDH was used as a loading control. **b** Quantification of the expression of the proteins shows significantly decreased levels in heat treatment groups. Data are presented as mean ± SEM, *n* = 3. *, *P* < 0.05. **c-d** Western blot analysis showing AKAP4, ODF1, ODF2, GAPDHS, SPESP1, and ACTRT2 proteins expression from mouse sperms of control and heat treatment (**c**) and the quantification of these proteins levels in (**d**). Data are presented as mean ± SEM, n = 3. *, P < 0.05

To validate the proteomics finding, the proteins related to sperm motility and energy metabolism AKAP4, SPESP1, ODF1, ODF2, GAPDHS, and ACTRT2 were evaluated in human sperms by western blotting. In line with our proteomics results, the proteins levels of sperm motility and energy metabolism-related proteins AKAP4, SPESP1, ODF1, ODF2, GAPDHS, and ACTRT2 were all significantly decreased in human sperms after heat treatment in comparison with control sperms (Fig. 4a-b), indicating that the proteomic reported here was reliable. Meanwhile, we operated mice subjected to testicular warming in a 43 °C or 33 °C water bath for 30 min once time. After 21 days, epididymis sperm samples were collected and used for western blotting. Meaningfully, these proteins AKAP4, ODF1, ODF2, GAPDHS, SPESP1, and ACTRT2 were down-regulated in mouse sperms of heat treatment compared with control (Fig. 4c-d). Collectively, these results indicated that these proteins AKAP4, ODF1, ODF2, GAPDHS, SPESP1, and ACTRT2 were significantly down-regulated after heat treatment of scrotum, suggesting that these proteins may serve as the biomarkers for scrotal heat treatment-induced male infertility.

## Discussion

In the study, we identified 61 proteins significantly deregulated in human sperms of 6 weeks after first heat treatment of the scrotum compared with control using iTRAQ and 2D-LC–MS/MS and selected some proteins related to sperm motility and energy metabolism for verification by western blotting in human and mouse sperms.

These deregulated proteins were found to be involved in several biological processes, including sexual reproduction, fertilization, gametogenesis, sperm motility, cell development, and sperm differentiation. The MF enrichment analysis suggests that the identified differentially expressed proteins are mainly involved in nucleotide binding and nucleoside phosphate binding, consistent with the main functions of these significantly change proteins reported in the previous proteomics study of local heat shock to the scrotum [21]. Our results revealed some new proteins associated with sperm motility, that might be transcribed completely before the metaphase of spermatogenesis and translated in spermatozoa, for example, AKAP4 and mitochondrial-encoded proteins [29].

It takes approximately 64 days in humans and 34.5 days in mice from the first spermatogenesis wave to sperm, and both require four spermatogenic cycles, of which latter 3/4 interval of the four spermatogenic cycles prepares the stage VII spermatids to form mature spermatozoa, which move to the epididymis [28, 30]. In humans, transient scrotal hyperthermia induced the lowest sperm concentration and motility in the latter 3/4 cycle of the four spermatogenic cycles, i.e., at 6 to 8 weeks after heat shock [25]. The sperm concentration and motility of mice after brief scrotal heat treatment have been reported to be lowest at approximately 21 days after transient scrotal heat [28]. The time of lowest sperm concentration and motility after transient scrotal hyperthermia in humans can be considered equivalent to that in mice.

Of these deregulated proteins, most of the upregulated proteins bind with poly-tail RNA, nucleotides, and proteins and participate in biological processes such as mRNA processing and gene expression. Whereas, the down-regulated proteins were mainly involved in fructose transmembrane transport, fructose metabolism to pyruvate, and cell growth and development. We found some down-regulated proteins, such as AKAP4, GAPDHS, SPESP1, ACTRT2, ODF2, and ODF1, play an important role in spermatogenesis. AKAP4 was mainly expressed in the tail of spermatozoa [31] and is involved in sperm motility. It acted as a signaling molecule to regulate spermatozoa and mature sperm acrosome activation by stimulating a signaling cascade [32–34], which was essential for sperm motility and fertility [35]. Heat treatment induces sperm DNA fragmentation significantly increasing and spermatozoa apoptosis [25, 36, 37]. Furthermore, knockdown of AKAP4 led to increased DNA fragmentation and apoptosis [38]. That indicated AKAP4 downregulation is associated with human sperm damage after the transient scrotal hyperthermia. GAPDHS was a testis-specific enzyme expressed at the tubular fibrous sheath of the main segment of the sperm flagellum. It participates in glycolysis and glycogen metabolism pathways and the synthesis of ATP, which is important for sperm motility. *Gapdhs* deficient mice exhibited non-motile sperm and male sterility [39, 40]. Abnormal GAPDHS expression led to non-obstructive azoospermia [41]. SPESP1 is involved in the fusion of sperm and egg cells and its abnormal expression leads to abnormal distribution of other proteins in spermatozoa. Meanwhile, *Spesp1* mutant mice have been shown to low sperm viability and male infertility [42, 43]. The cytoskeletal protein ACTRT2 is expressed at the post-acrosomal region and middle part of human sperm and plays a role in sperm motility. ACTRT2 expression in the mature spermatozoa of patients with varicocele was previously shown to be up-regulated [44], in contrast to the results of the ACTRT2 expression obtained in our study. This difference suggests that the short-term heat shock of scrotum causes its upregulation for compensation. ACTRT2 expression has not been evaluated in mice or other species. Our PPI analysis revealed an interaction between ACTRT2 and GAPDHS, but the specific functions of these proteins remain to be clarified. Abnormal ODF1 expression has been shown to cause abnormal sperm morphology and low sperm motility [45]. ODF1, also known as HSP10, plays an important role in sperm head and neck connections and *Odf1* defect mice have been shown to exhibit impairment in the mitochondrial sheath movement of spermatozoa [46, 47]. Previous studies have demonstrated that ODF2 transcription was mainly initiated in primary spermatocyte and, in addition to ODF2 post-processing, combination, and storage is regulated by the hnRNP (A, M, U, K) family proteins [48, 49]. It is then expressed in round spermatids, which interacts with Tssk4 to regulate sperm movement [50]. Thus, these deregulated proteins are closely associated with low sperm viability and concentration after the heat treatment of scrotum and reversible male infertility.

In summary, we used iTRAQ and 2D-LC–MS/MS to identify differential protein expression profiles in human sperms from control and heat treatment of scrotum and found the expression of 61 sperm proteins deregulated after transient scrotal hyperthermia, which consequently decrease sperm concentration and motility. These proteins were mainly involved in sexual reproduction, fertilization, sperm motility, and cellular development. Besides, these proteins AKAP4, ODF1, ODF2, GAPDHS, SPESP1, and ACTRT2 were examined to better verify these results in mice after heat treatment. Further studies of the relationship between these proteins and heat treatment of scrotum are needed to confirm and to further dissect the mechanisms underlying, which are promising to be the biomarkers and clinical targets for scrotal heat treatment-induced male infertility.

## Supplementary information

**Additional file 1.**

**Additional file 2.**

## Data Availability

The datasets used and/or analyzed during the current study are available from the corresponding author on reasonable request.

## Abbreviations

iTRAQ: isobaric Tags for Relative and Absolute Quantitation
2D-LC-MS/MS: Two-dimensional liquid chromatography–tandem mass spectrometry
AKAP4: A-Kinase anchor protein 4
ODF2: Outer dense fiber protein 2
ODF1: Outer dense fiber protein 1
SPESP1: Sperm equatorial segment protein 1
ACTRT2: Actin-related protein t2
GO: Gene ontology
hnRNP: Heterogeneous Nuclear Ribonucleoprotein GAPDHS, Glyceraldehyde-3-Phosphate dehydrogenase, testis-specific
FDR: False discovery rate
